# Feasibility of an Automatic Ultrasonographic Image Acquisition System Associated With an Artificial Intelligence Algorithm for Real-Time Monitoring of Cardiac Motion During Cardiac Radio-Ablation

**DOI:** 10.3389/fcvm.2022.849234

**Published:** 2022-04-25

**Authors:** Matteo Casula, Veronica Dusi, Saskia Camps, Jérémie Gringet, Tristan Benoit, Adriano Garonna, Roberto Rordorf

**Affiliations:** ^1^Arrhythmia and Electrophysiology Unit, Fondazione IRCCS Policlinico San Matteo, Pavia, Italy; ^2^Section of Cardiology, Department of Molecular Medicine, University of Pavia, Pavia, Italy; ^3^Unit of Cardiology, AOU Città della Salute e della Scienza, Turin, Italy; ^4^EBAMed SA, Geneva, Switzerland

**Keywords:** cardiac radioablation, motion monitoring, ventricular arrhythmia, echocardiography, artificial intelligence

## Abstract

**Background:**

The management of the cardio-respiratory motion of the target and the reduction of the uncertainties related to patient's positioning are two of the main challenges that stereotactic arrhythmia radio-ablation (STAR) has to overcome. A prototype of a system was developed that can automatically acquire and interpret echocardiographic images using an artificial intelligence (AI) algorithm to calculate cardiac displacement in real-time.

**Methods:**

We conducted a single center study enrolling consecutive patients with a history of ventricular arrhythmias (VA) in order to evaluate the feasibility of this automatic acquisition system. Echocardiographic images were automatically acquired from the parasternal and apical views with a dedicated probe. The system was designed to hold the probe fixed to the chest in the supine position during both free-breathing and short expiratory breath-hold sequences, to simulate STAR treatment. The primary endpoint was the percentage of patients reaching a score ≥2 in a multi-parametric assessment evaluating the quality of automatically acquired images. Moreover, we investigated the potential impact of clinical and demographic characteristics on achieving the primary endpoint.

**Results:**

We enrolled 24 patients (63 ± 14 years, 21% females). All of them had a history of VA and 21 (88%) had an ICD. Eight patients (33%) had coronary artery disease, 12 (50%) had non-ischemic cardiomyopathy, and 3 had idiopathic VA. Parasternal, as well as apical images were obtained from all patients except from one, in whom parasternal view could not be collected due to the patient's inability to maintain the supine position. The primary endpoint was achieved in 23 patients (96%) for the apical view, in 20 patients (87%) for the parasternal view, and in all patients in at least one of the two views. The images' quality was maximal (i.e., score = 4) in at least one of the two windows in 19 patients (79%). Atrial fibrillation arrhythmia was the only clinical characteristics associated with a poor score outcome in both imaging windows (apical *p* = 0.022, parasternal *p* = 0.014).

**Conclusions:**

These results provide the proof-of-concept for the feasibility of an automatic ultrasonographic image acquisition system associated with an AI algorithm for real-time monitoring of cardiac motion in patients with a history of VA.

## Introduction

The therapeutic strategies currently available for the prevention of ventricular arrhythmias (VAs), namely antiarrhythmic drugs and invasive catheter ablation, are limited by suboptimal efficacy and a non-negligible incidence of adverse events and procedural complications (1–4). Furthermore, some arrhythmic patients with refractory VAs, are not eligible for traditional invasive ablative approaches due to their frailty and/or the inability to access VAs substrate with catheters (5). With the aim to offer a further therapeutic strategy for these patients, the possibility of treating arrhythmias was devised and developed by delivering high dose of ionizing radiations focused on the tissues critical for the genesis of arrhythmias [i.e., stereotactic arrhythmia radio-ablation (STAR)] (6, 7). The clinical experiences accumulated so far in this field have shown that the management of the cardio-respiratory movements of the target and the reduction of uncertainties related to patient positioning are two critical challenges that STAR has to overcome (8). The need for target's movement management is of the utmost importance particularly in case of respiratory gated delivery for radiotherapy with heavy particles such as protons and carbon ions (9, 10). At present, the strategies applied for cardio-respiratory movements compensation are limited by the need to consistently increase the size of the treated volume (e.g., internal target volume generated by 4D cardiac or respiratory CT or both), extend treatment time (e.g., gated delivery), and globally by the unsolved need to directly monitor cardio-respiratory movements in real-time without the use of fiducial markers (6, 8, 11, 12). A possible solution to this issue could be represented by the use of echocardiography as a fully non-invasive tool for monitoring internal motion. However, the context of radiotherapy treatment offers new challenges even for this versatile tool, such as the need for an immobilization system for the probe and the need for an automatic acquisition system that works in supine position and is able to process the acquired images with extremely short computation times and provide precise information about cardiac movements. A prototype of a system was developed that can automatically acquire and interpret echocardiographic images using an artificial intelligence (AI) algorithm to calculate cardiac displacement in real-time (EBAMed SA, Geneva, Switzerland). The development and the first experiments of this system were carried out on a general cardiology patient database (13) and on healthy volunteers; moreover, the set of images on which the algorithm was trained consisted of echocardiographic sequences mostly acquired in left lateral decubitus. No previous studies have evaluated the feasibility of this system in the context and on the patient population for which it was designed. Therefore, the aim of this study was to evaluate the ability of the automatic echocardiographic imaging system to obtain images of sufficient quality to be correctly interpreted by the AI algorithm in patients with a history of VAs in supine position, as well as to identify any factors limiting acquisition in this specific setting and population.

## Materials and Methods

We conducted a single center, single arm, feasibility study on patients referred to the Arrhythmia and Electrophysiology Unit of the Fondazione IRCCS Policlinico San Matteo, Pavia, Italy, with a previous history of VAs. All consecutive patients evaluated in our clinic between May and September 2021 were screened for enrollment. This study received ethical approval from the local institutional review board (approval number 57629/2021) and, after being properly informed, all participants signed a written informed consent.

### The Acquisition System

**Figure 1:**
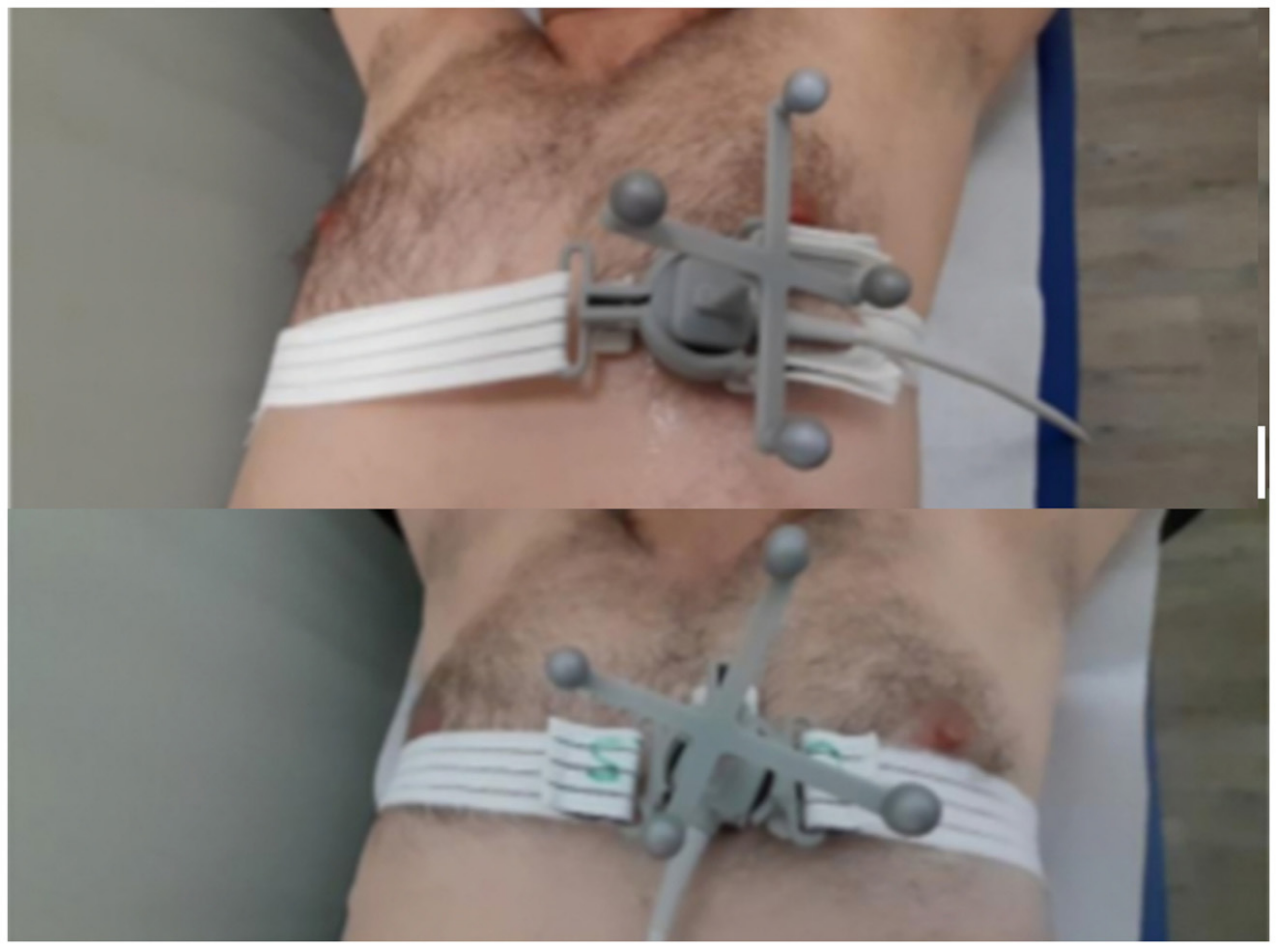
Images of the ultrasound probe housed in the holder containing the markers for optical localization. The probe and the support are kept adherent to the patient's chest by means of an adjustable elastic band. Upper panel apical position; Lower panel parasternal position.

**Figure 2:**
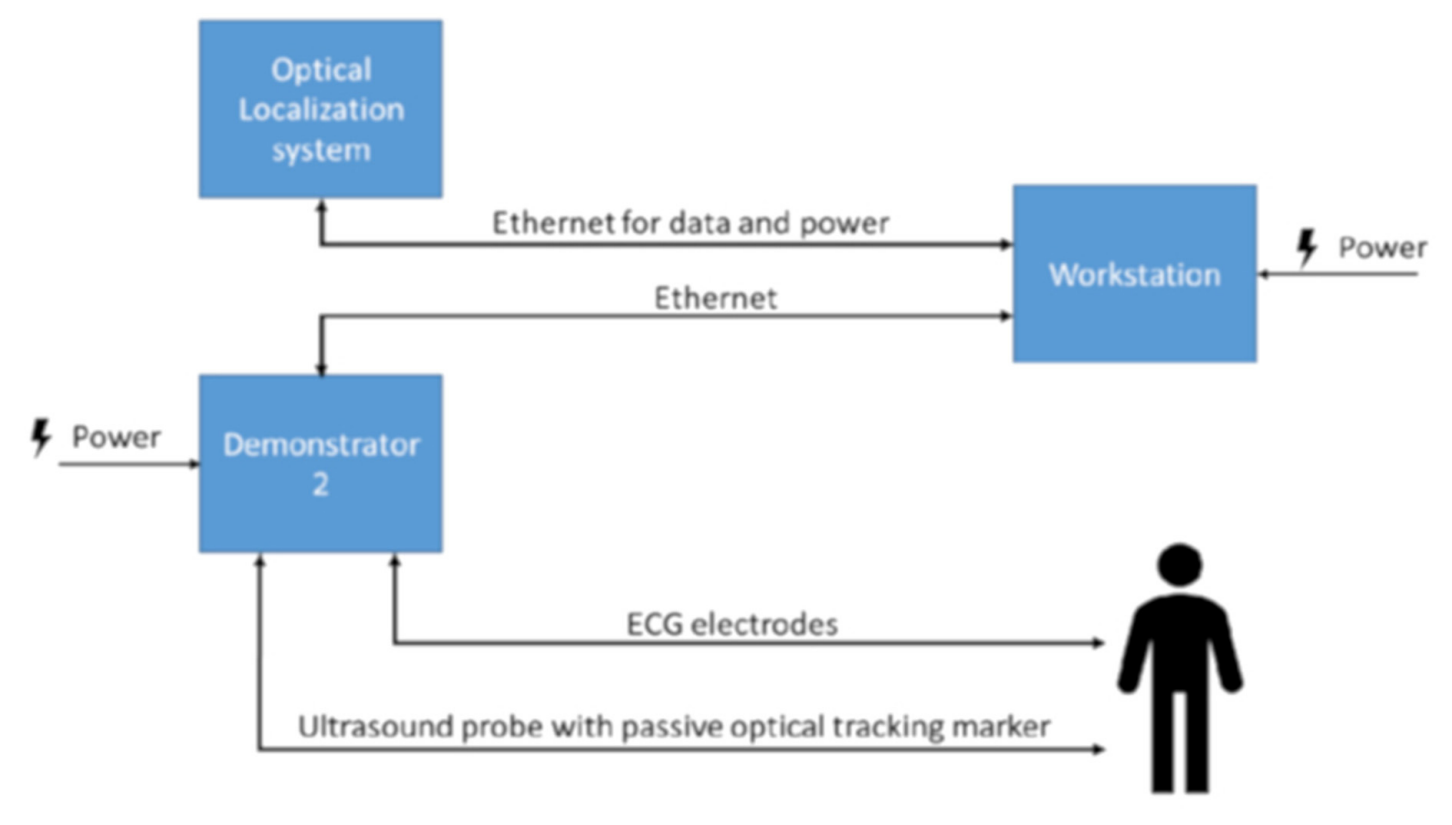
Scheme representing of the acquisition system flow.

The image acquisition system used in the study consisted of a dedicated echocardiographic probe positioned inside a support and held adherent to the patient's chest by means of an adjustable elastic band. Two types of holders were conceived, alternatively used for the acquisitions made from the apical and parasternal windows (Figure 1). Each of the two holders housed four spherical references functional to an optical location system of the probe position (Polaris Vega® XT, NDI, Ontario, Canada). Simultaneously with the acquisition of echocardiographic images, the surface electrocardiographic signal (ECG) was recorded through three adhesive electrodes positioned at the root of both upper limbs and at the level of the left antero-superior iliac spine. The R-waves were automatically detected by the AccuSync® 42 trigger (AccuSync Medical Research Corporation, Milford, CT, USA). The acquired echocardiographic and ECG signals were conveyed to the processing module called Demonstrator 2 developed by EBAMed SA (Geneva, Switzerland). Beamforming of echocardiographic signals was performed using a Terason USB3.0 Engine (Teratech Corporation, Boston, MA, USA). The ultrasounds system recorded bidimensional (B-mode) ultrasound images at 40 Hz from two perpendicular plans. Once processed, the data were sent to the workstation which communicated with the Demonstrator 2 and with the optical localization system of the probe and provided a graphical interface for the operator (Figure 2). The interface screen showed in real time the ECG trace and echocardiographic images, as well as information on the position of the probe and any alarms. During the imaging session, the patients were asked to assume the supine position with the head resting on a suitable support and, when tolerated, to keep the arms raised.

### Images Interpretation From the AI Algorithm

**Figure 3:**
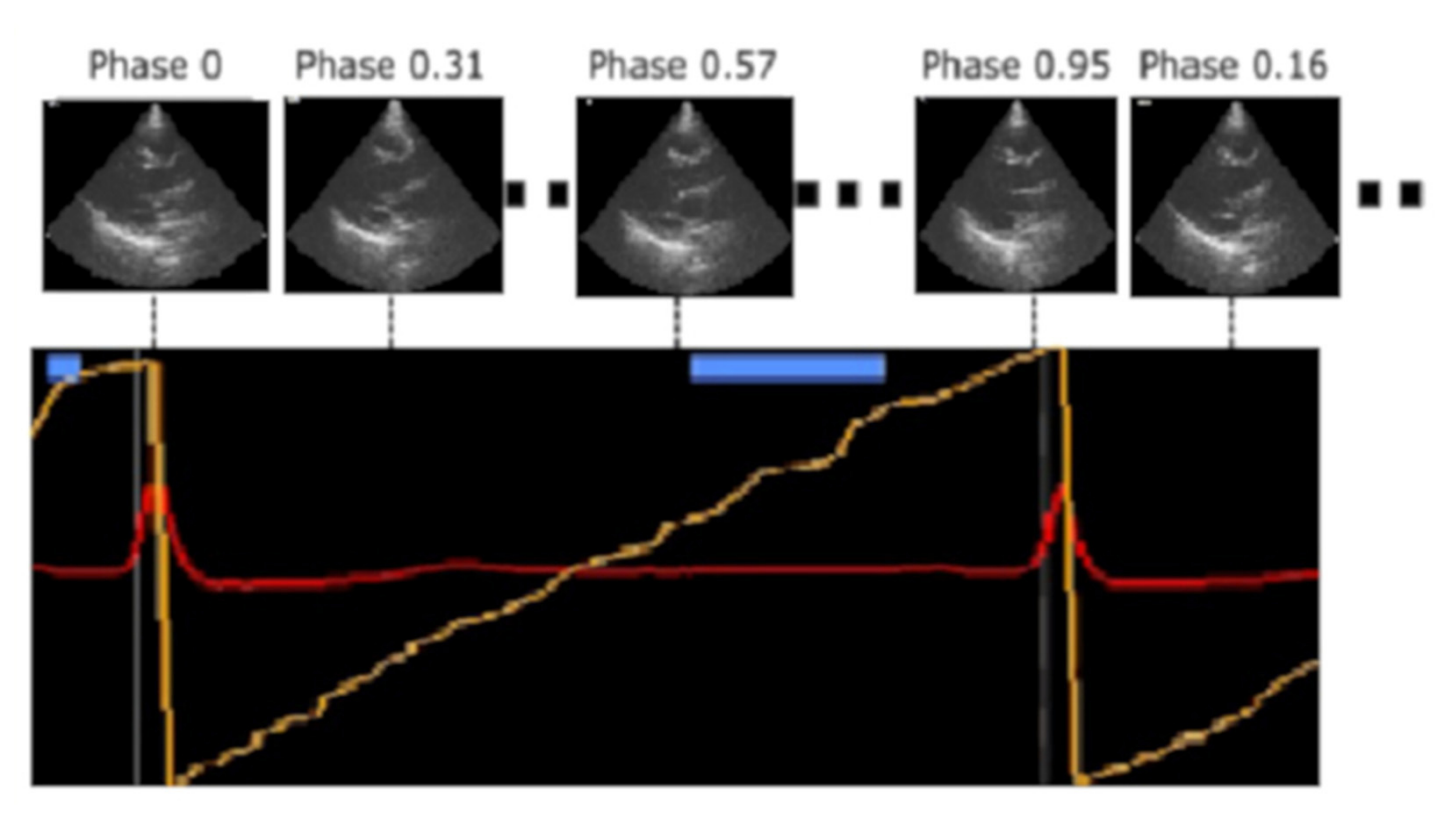
Identification of the cardiac cycle phase performed by the artificial intelligence algorithm through the real-time analysis of the acquired ultrasound images. A linear mapping between 0 and 1 (yellow line) was performed in the R-R peak interval and a cardiac phase was assigned to each ultrasound frame based on its temporal position within this interval.

The data acquired with the described instrumentation were processed by an AI algorithm previously developed by EBAMed SA, (Geneva, Switzerland) capable of identifying the phase of the corresponding cardiac cycle for each ultrasound image acquired and calculating the extent of the displacement of the image compared to an image acquired in the same phase of a reference cardiac cycle. To obtain the ground truth cardiac phases, a phase of 0 was assigned to each R-peak in the ECG trace and a linear mapping between 0 (included) and 1 (excluded) of the remaining cardiac phases in the R-R peak interval was performed. A cardiac phase was assigned to each ultrasound frame based on its temporal position within the interval (Figure 3).

**Figure 4:**
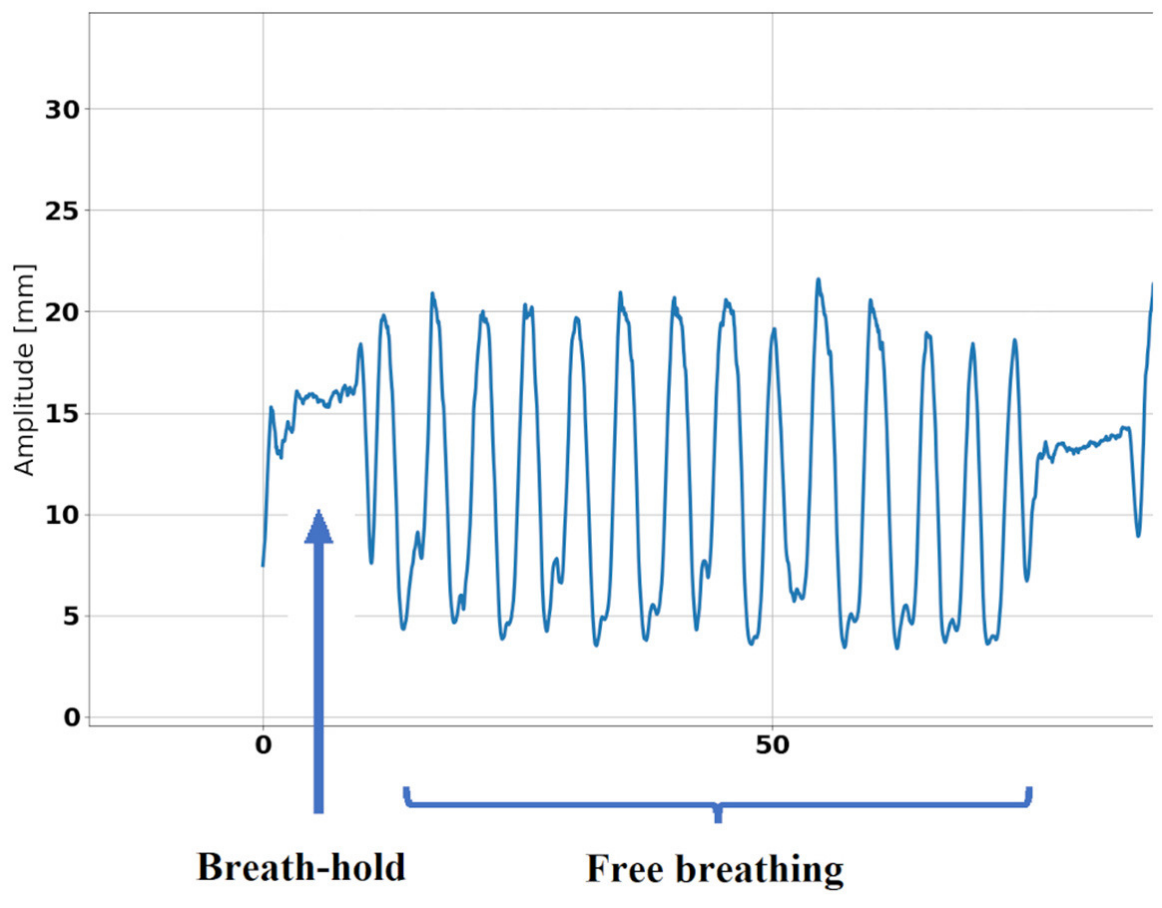
Example of heart displacement measured by ultrasound during respiratory exercise.

The AI algorithm used for cardiac phase identification is based on a neural network which consists of two parts. The first part, a multi-stage three-dimensional (3D) causal convolution network, is responsible for the extraction of spatial and short-term temporal features from the ultrasound sequence. The second part, a single dimension (1D) temporal convolution neural network, extracts long term temporal features. The network takes an ultrasound sequence of an arbitrary length as input, and it outputs one cardiac phase for each ultrasound image in the sequence. A publicly available database which contains US sequences and ECG traces of 500 cardiac patients (14) was used for the network training and evaluation using 5-fold cross validation. Once the cardiac phase is determined, a separate and additional neural network, previously developed by EBAMed SA, is used to measure the heart displacement in three dimensions (see Figure 4). This neural network is inspired by work of de Vos et al. (15) and it determines the heart displacement using rigid registration between the real-time ultrasound image and the reference ultrasound image (for the same cardiac phase). After inputting the real-time and reference ultrasound images, they are concatenated and subsequently passed through several convolution blocks followed by feature map averaging. Subsequently, three paths of fully-connected layers output a rotation angle, as well as a translation in two directions for each perpendicular ultrasound plane. As the location of each (heart) pixel inside the images is known in 3D space thanks to the optical localization system, the output of the network can be used to provide the displacement of the heart in 3D space.

### The Acquisition Protocol

During the acquisition of the images, the patients were asked to remain with the chest completely uncovered and to assume the supine position on the acquisition table, with the head resting on a special support and, when tolerated, keeping their arms raised. The ECG cables were positioned as described above and, after applying the gel, the ultrasound probe was positioned and fixed using the appropriate holder and an elastic tape, at the level of the apical echocardiographic acquisition window. The position of the probe on the chest was noted on the case report form (CRF) and the relative position was monitored through the optical tracking system. Once the positioning of the patient and the initialization of the computer systems were completed, the monitoring of the heart position began. In the first 5 min of acquisition, the patients were asked to relax and breathe normally (*free breathing*); over the next 5 min they were encouraged to take a deep exhalation and hold their breath for 10 s every min, 5 times (*respiratory exercise*). At the end of this phase the probe was removed and repositioned at the level of the parasternal window. The position of the probe in the chest was noted in the CRF and the acquisition procedure was repeated. At the end of the acquisition phase, the probe was removed, and the patients were allowed to clean themselves of the gel left on the chest. Subsequently, before leaving, patients were asked to report any discomfort experienced during the procedure. The described acquisition protocol was conducted by a team of clinicians from the IRCCS San Matteo of Pavia with experience in the field of echocardiography and technicians from the EBAMed SA company.

### Population

Screened patients were eligible for the enrollment if they had a history of VAs, were at least 18 years old, were able to maintain the supine position for the time of acquisition, did not have an ongoing VAs, and agreed to be enrolled in this study.

### Outcomes

The primary endpoint was defined as the percentage of patients able to obtain a positive result in a multi parametric score of image quality, consisting of:

A– Image quality in terms of allowing a correct identification of the phase of the cardiac cycle by the prototype software:
  Score = 1: average phase error per patient, defined as the difference between the phase of the cardiac cycle identified by the algorithm and the one evaluated by the ECG reference <0.1.
  Score = 0: average phase error per patient ≥0.1.
B– Image quality in terms of allowing a correct measurement of the heart displacement (mainly due to respiratory motion) by the prototype software:
  Score = 1: maximum excursion calculated by the algorithm <30 mm with a total 3D error in the calculation of the displacement <3 mm.
  Score = 0: either maximum excursion calculated by the algorithm ≥30 mm or total 3D error in the calculation of the displacement ≥3 mm (or both).
C– Image quality in terms of the ability to distinguish typical cardiac structures, as assessed visually by the clinical operator:
  Score = 1: ability to identify visually by an experienced operator in the acquired image at least one of the following structures: left ventricular free wall, interventricular septum, mitral valve, or aortic valve.
  Score = 0: inability to identify at least one of these structures.
D– Image quality in terms of the stability of the image throughout the respiration cycle, as assessed visually by the clinical operator:
  Score = 1: persistence of cardiac structures within the echocardiographic image during respiratory motion.
  Score = 0: disappearance of cardiac structures from the echocardiographic image during respiratory motion.

For each patient, scoring was done for each imaging view (i.e., parasternal and apical). If the score was 2 (at least 1 point in A or B and 1 point in C or D) or greater for at least one of the imaging views, the outcome was considered as positive. The final result is the proportion of patients (in %) with a positive outcome, defined as the number of patients with a positive evaluation divided by the total number of patients x 100.

The secondary endpoints of the study were the percentage of patients able to obtain a positive result in each of the items of the primary endpoint and the percentage of patients with maximum image quality for algorithm operation, defined as those patients who scored a 4 on the multi-parametric assessment.

Scores A, C, and D were evaluated on *free-breathing* sequences while score B was evaluated during *respiratory exercise*. The reference for calculating the phase error of the cardiac cycle was the ECG signal acquired simultaneously with the ultrasonographic images. The magnitude of the maximum accepted error was set to 0.1 consistent with the performance obtained by the algorithm on the validation dataset. Prior to assignment of score A and B, ECG traces acquired with their associated automatic R-wave markers were reviewed by an experienced operator and any inconsistencies between the automatic marker and the operator's opinion were recorded in a special log. Markers referable to ventricular and supraventricular premature complexes were also identified. Images acquired during extrasystolic cycles or during those in which the automatic markers were not consistent with the operator's opinion were excluded from scores A and B analyses. Regarding the tracking of heart displacement, the maximum acceptable threshold in the displacement calculated by the algorithm was conservatively set at 30 mm, in order to exclude that the magnitude of this excursion was not consistent with the maximum displacement of a cardiac tissue reported in the literature. The total geometric error in 3D space in the calculation of position was taken as the relevant metric. The threshold value of 3 mm was calculated as 10% of the maximum accepted excursion, consistent with the performance obtained by the algorithm on the validation dataset.

### Statistics

#### Sample Size

As this is a feasibility study at an early stage of research, an enrollment of 24 patients was planned. With the goal of obtaining 90% positive patients at the primary endpoint (success in 22 out of 24 patients), enrollment of this number of patients would ensure a 95% confidence interval between 71 and 98%.

#### Data Analysis and Presentation

Outcomes were reported as the number of patients who achieved the outcome with the relative percentage and 95% confidence interval (CI). The impact of clinical and demographic characteristics of the enrolled patients on the quality of the acquired images was also assessed. The descriptive variables collected were presented as number and relative percentage for categorical variables and as mean ± standard deviation or median (interquartile range) for continuous variables, as appropriate based on the normality of the distribution of the variable in question verified by Shapiro-Wilk test. Comparisons between means were performed with the *t*-test or the Welch-test, based on the result of the F-test previously performed to compare the variances between groups. Comparisons between medians were made with the Mann-Whitney test and categorical variables were compared with the Chi^2^ test or Fisher's exact test, as appropriate. Patients who experienced significant protocol violations were excluded from the analysis, as detailed in the next sections.

## Results

### Population Characteristics

During the period from May 2021 to September 2021, 24 patients were enrolled in the study. Five were female (21%). The mean age of the patients was 63 ± 14 years. All patients had a history of at least one episode of VA: in 23 patients (96%) at least one ventricular tachycardia (VT) had been recorded and 4 patients (17%) had at least one episode of ventricular fibrillation (VF). Most of the enrolled patients had an ICD (87.5%) and 3 patients were monitored with a loop-recorder (12.5%). The etiology of the arrhythmia was ischemic in 8 patients (33%), 12 patients (50%) had non-ischemic cardiomyopathy, and 3 patients had a history of idiopathic VT/VF. Further details on the characteristics of the enrolled population are presented in Table 1.

**Table 1:** Characteristics of the enrolled population.

	**Number of patients enrolled**	**24**
Clinical and demographics characteristics	Age (years)	63 ± 14
	Female gender	5 (21%)
	Height (cm)	173 ± 7
	Weight (kg)	82 ± 16
	BMI (kg/m^2^)	26 (24–30)
	Left ventricular ejection fraction (%)	52.5 (36.5–60)
	History of smoking	17 (71%)
	COPD or other significant pneumopathy	6 (25%)
History of arrhythmias	History of VT	23 (96%)
	History of VF	4 (17%)
	History of atrial arrhythmias	8 (33%)
	Previous VT ablation	9 (38%)
Type of heart disease	Ischemic heart disease	8 (33.3%)
	Non-ischemic cardiomyopathy	12 (50%)
	Dilated cardiomyopathy	4 (16.6%)
	Hypertrophic cardiomyopathy	4 (16.6%)
	Arrhythmogenic cardiomyopathy	1 (4.2%)
	Other cardiomyopathies	3 (12.5%)
	Corrected congenital heart disease	1 (4.2%)
	Absence of structural heart disease	3 (12.5%)
Devices	ICD	21 (87.5%)
	Single-chamber ICD	7 (29%)
	Dual-chamber ICD	4 (17%)
	Biventricular ICD	7 (29%)
	Subcutaneous ICD	3 (12.5%)
	Loop recorder	3 (12.5%)
Mechanical Valve		1 (4%)

*Data are presented as number (%), mean ± standard deviation or median (interquartile range); BMI, body mass index, COPD, chronic obstructive pulmonary disease; ICD, implantable cardioverter defibrillator; VF, ventricular fibrillation; VT, ventricular tachycardia*.

### Acquisitions

The mean heart rate (HR) during acquisitions was 63 ± 8 bpm for the apical window and 62 ± 8 bpm for the parasternal window. Two patients (8%) had an irregular rhythm due to atrial fibrillation throughout the acquisition, and 6 (25%) patients had an extrasystolic burden, defined as the percentage of extrasystolic atrial or ventricular complexes on total complexes, >10%.

### Deviations

Both imaging views were attempted in all patients except one who, after having performed the respiratory exercise for the apical window acquisition, developed an access of cough that made it impossible to continue the experiment with the acquisition from the parasternal window. This patient was therefore excluded from the score evaluation for the parasternal window. In two patients it was not possible to obtain, despite repeated attempts, a parasternal view adequate for image acquisition. For these two patients it was therefore not possible to calculate the performance of the algorithm for points A and B, and a score of 0 was assigned in points C and D; consequently, it was considered that these patients did not obtain a positive evaluation in the multiparametric score of the primary outcome.

In one case it was not possible to obtain an apical window from which the cardiac structures did not disappear from the ultrasound image during the respiratory exercise. This patient was therefore excluded from the evaluation of score B for the apical window.

Because of a not always optimal quality of the ECG trace during acquisition, at least one oversensing phenomenon of deflections different from R-wave occurred in 9 patients (37.5%) and at least one episode of R-wave undersensing occurred in 7 (29%) patients. Images acquired during extrasystolic cycles and during those for which the ECG trace was subject to undersensing or oversensing errors, thus not being able to be used as a reference of the cardiac cycle phase, were excluded from the analyses of score A and B.

### Outcomes

The primary outcome was achieved in 23 patients (96%, CI 95% 79–100%) for the apical window, in 20 patients (87%, CI 95% 66–97%) for the parasternal window, and in all patients (100%, CI 95% 86–100%) in at least one of the two windows (Table 2).

**Table 2:** Multi-parametric score results and primary outcome.

**Scores**	**Apical view**	**Parasternal view**	
A-average cardiac phase error <0.1	20 out of 24 (83%, CI 95% 62–95%)	18 out of 21 (86%, CI 95% 64–97%)	
B-maximum excursion <30 mm and total displacement error <3 mm	22 out of 23 (96%, CI 95% 79–100%)	20 out of 21 (95%, CI 95% 76–100%)	
C-ability to visually identify cardiac structures	23 out of 24 (96%, CI 95% 79–100%)	20 out of 23 (87%, CI 95% 66–97%)	
D-persistence of cardiac structures in the image during breathing	22 out of 24 (92%, CI 95% 73–99%)	18 out of 23 (78%, CI 95% 56-92%)	**At least one view**
Primary outcome (score ≥2 with at least 1 within A and B and at least 1 within C and D)	**23 out of 24** **(96%, CI 95% 79–100%)**	**20 out of 23** **(87%, CI 95% 66–97%)**	**24 out of 24** **(100%, CI 95% 86–100%)**

*CI, confidence interval*.

A mean phase error in the correct identification of the cardiac cycle phase <0.1 was found in 20 patients (83%, CI 95% 62–95%) for images acquired from the apical window and in 18 patients (86%, CI 95% 64–97%) for the parasternal one. The average phase error was 0.05 ± 0.04 and 0.06 ± 0.06, respectively for the apical and parasternal windows (Table 3).

**Table 3:** Additional detailed results for scores A and B.

		**Apical**	**Parasternal**
A	Average phase error [threshold value used in score A is 0.1]	0.05 ± 0.04	0.06 ± 0.06
B	Respiratory motion amplitude (mm) [threshold value used in score B is 30 mm]	17 ± 7	16 ± 8
	3D Error in calculation of displacement (mm) [threshold value used in score B is 3 mm]	1.1 ± 0.2	1.1 ± 0.4

*Data are presented as mean ± standard deviation*.

A cardiac displacement >30 mm was measured for one patient in apical view (31 mm) and another patient in parasternal view (31 mm). On average, recorded displacements were 17 ± 7 mm in apical view and 16 ± 8 mm in parasternal view, with an average 3D error of 1.1 ± 0.2 mm and 1.1 ± 0.4 mm, respectively.

In 23 patients (96%, CI 95% 79–100%) for the apical window and in 20 for the parasternal one (87%, CI 95% 66–97%) it was possible to identify by an experienced operator at least one among the free wall of the left ventricle, the interventricular septum, or a valvular structure. For two patients it was not possible to obtain, despite repeated attempts, a parasternal window sufficient for the identification of these structures.

In 2 patients for the apical view (8%, CI 95% 1–27%) and in 5 for the parasternal window (22%, CI 95% 8–44%) cardiac structures transiently disappeared from the echocardiographic image during the quiet breathing movement.

The image quality scores were maximal (with a multiparametric score of 4), in 16 patients for both apical and parasternal (70%, CI 95% 48–87%) views, and in 19 patients (79%, CI 95% 56–93%) in at least one of the two windows.

During the procedures related to the experimental protocol, no significant adverse events occurred to the patients. Only one patient, with advanced chronic heart failure and smoke-induced chronic obstructive pulmonary disease, had to stop the experiment due to a coughing access following the effort made to perform the respiratory exercise during the acquisition of the apical window.

### Influence of Parameters on Results

When analyzing the clinical characteristics of patients, the only parameters found to be statistically correlated with the achievement of images of maximal quality were the heart rate and heart rhythm stability during acquisition (Tables 4, 5). Irregular heart rhythm due to atrial fibrillation resulted in higher median errors for the cardiac cycle phase identification (0.13 vs. 0.03, *p* = 0.0215 for the apical view, and 0.11 vs. 0.03, *p* = 0.0381 for the parasternal view). Excluding patients with atrial fibrillation arrhythmia from the analysis, no statistically significant differences were observed between the heart rate of the patients who obtained a positive evaluation on score A compared to those who did not (62 ± 7 vs. 63 ± 4, *p* = 0,83). Notably, as previously mentioned, images acquired during extrasystolic cycles were excluded from scores A and B analyses. Accordingly, no differences were found based on extrasystolic burden (Table 4).

**Table 4:** Comparison of the clinical demographic characteristics of patients with maximal image quality versus those with lower image quality.

	**Patients with maximal image quality in at least one ultrasound window** ***N = 19 (79%, CI 95% 56–93%)***	**Patients with suboptimal image quality in both ultrasound windows** ***N = 5 (21%, IC 95% 7–42%)***	***P*-value**
Age (years)	62 ± 15	68 ± 6	0.38
Female gender	5 (26.3%)	0 (0%)	0.54
Height (cm)	172 ± 7	174 ± 8	0.55
Weight (kg)	80 ± 13	89 ± 25	0.27
BMI (kg/m^2^)	26 (24–29)	25 (23–35)	0.97
LV ejection fraction (%)	55 (36–60)	46 (37–59)	0.72
History of smoking	13 (68.4%)	4 (80%)	1
COPD	5 (26.3%)	1 (20%)	1
History of VT	19 (100%)	4 (80%)	0.21
History of VF	2 (10.5%)	2 (40%)	0.18
History of atrial arrhythmias	4 (21.1%)	4 (80%)	**0.03**
Previous VT ablation	5 (26.3%)	4 (80%)	0.05
Ischemic heart disease	7 (36.8%)	1 (20%)	0.63
Non-ischemic cardiomyopathy	9 (47.4%)	3 (60%)	1
Absence of structural heart disease	3 (15.8%)	0 (0%)	1
Single-chamber ICD	4 (21.1%)	3 (60%)	0,13
Dual-chamber ICD	3 (15.8%)	1 (20%)	1
Biventricular ICD	6 (31.6%)	1 (20%)	1
Subcutaneous ICD	3 (15.8%)	0 (0%)	1
Loop recorder	3 (15.8%)	0 (0%)	1
Mean HR (bpm)	61 ± 7	69 ± 7	**0.04**
Atrial fibrillation arrhythmia	0 (0%)	2 (40%)	**0.036**
Extrasystolic burden > 10%	5 (26.3%)	1 (20%)	1

*Data are presented as number (%), mean ± standard deviation or median (interquartile range); BMI, body mass index; bpm, beats per minute; CI, confidence interval; COPD, chronic obstructive pulmonary disease; HR, heart rate; ICD, implantable cardioverter defibrillator; VF, ventricular fibrillation; VT, ventricular tachycardia*.

**Table 5:** Evaluation of the impact of heart rate and heart rhythm stability on the ability of the algorithm to correctly identify the phase of the cardiac cycle.

		**Apical score**			**Parasternal score**		
		***1***	***0***	***P***	***1***	***0***	***P***
**A**	Mean HR during acquisition (bpm)	62 ± 7	71 ± 10	**0.048**	61 ± 8	69 ± 8	0.107
	Atrial fibrillation arrhythmia during acquisition	0 out of 20 (0%)	2 out of 4 (50%)	**0.022**	0 out of 18 (0%)	2 out of 3 (100%)	**0.014**

*Data are presented as mean ± standard deviation*.

## Discussion

The results of our study provide the proof-of-concept for the feasibility of an automatic ultrasound image acquisition system associated with an AI algorithm for real-time monitoring of heart movement in patients with a history of VAs.

As previously mentioned in the introduction, the need for target's movement management is of the utmost importance during arrhythmia radio-ablation and the strategies currently available for this task have several limitations. A possible solution could be the use of a relatively simple and cheap imaging system such as echocardiography. The additional advantages of an ultrasound-based motion management over current techniques are that the solution is fully non-invasive and enables real-time monitoring of the internal motion (as opposed to the use of external surrogates). The recently reported use of nuclear magnetic resonance imaging for this purpose is limited by the fact that a direct tracking of the heart as well as the heart's substructures was not possible (16). The context of radiotherapy treatment offers new challenges even for echocardiography. Obvious radiation protection requirements prevent a human operator from acquiring the ultrasound images during the delivery of therapy and force to develop automatic acquisition systems. The supine position assumed by the patient on the therapy table, not being for anatomical reasons the most suitable for the acquisition of echocardiographic images, makes this task even more difficult. Moreover, to be useful in the real-time guidance of treatment, the acquired images must be processed with extremely short computation times and provide precise information about cardiac movements. To try to meet these challenges, a prototype of a system for automatic acquisition of echocardiographic images was designed and developed (EBAMed SA, Geneva, Switzerland). The images thus acquired are then processed and interpreted by an AI algorithm to calculate the cardiac displacement in real time. The possibility to carry out this task, extremely complex for the common *rule-based* systems, is facilitated by the use of a technology based on *machine-learning* algorithms (17). The development and the first usages of this system were carried out on a general cardiology patient database (13) and on healthy volunteers, and the set of images on which the algorithm was trained consisted of echocardiographic sequences mostly acquired in left lateral decubitus. It is therefore necessary to test the feasibility of using this system in the context and on the patient population for which it was designed and which, as has been mentioned, proposes specific challenges. Accordingly, the main aim of the present study was to evaluate whether the automatic echocardiographic image acquisition system under study could obtain adequate images to ensure the functioning of the AI algorithm in real patients with a history of VAs and to identify any limiting factors for acquisition in this specific population. On the other hand, it was beyond the scope of this study to evaluate the functioning and reliability of the algorithm in tracking cardiac movements.

Some considerations can be made about the representativeness of the enrolled population compared to the population potentially eligible for radio-ablation treatment. The average age of the patients enrolled, as well as the percentage of females and the spectrum of underlying cardiac disorders, are globally in line with that of the types of patients who could benefit from STAR (6, 11, 18). All body sizes were coved, as well as all ranges of left ventricle ejection fraction, including patients with a markedly depressed left ventricle systolic function, that are at present the main candidates for STAR (6). Previous clinical studies on STAR did not systematically report on cardiac rhythm stability during treatment, however it appears plausible, even considering the percentage of patients with dual-chamber ICD and CRT (18), that the population included in our study, even in this respect, was representative of the cohort of patients eligible for STAR.

The primary endpoint of the study was achieved in all patients for at least one of the two windows. The attempt to acquire the parasternal window failed in 2 out of 23 patients, but in those patients in whom the window was obtained, there were no significant differences in terms of the quality of the images obtained compared with the apical window. The difficulty in acquiring the parasternal window can be partially explained by the supine position of the patient and the need to apply strong pressure of the probe on the thorax to obtain an image. However, considering that in a possible treatment phase the best of the two probe positions studied could be used, our results are reassuring.

The evaluation of the adequacy of the images provided by the automatic acquisition system for the definition of the phase of the cardiac cycle showed a good performance of the system in 83 and 86% of patients for the apical and parasternal windows respectively. The good quality of the acquired images is confirmed by the low average phase errors calculated, that are globally consistent with that showed by the algorithm on the validation set, thus confirming a good performance of the algorithm on real patients in the treatment position. For this score, as opposed to score B, the study conducted allows us to evaluate not only the quality of the images acquired but also the actual operation of the algorithm. Having available a known reference of the measured quantity (i.e., the phase of the cardiac cycle provided by the ECG hardware) the calculated average phase error can be considered as a real error. Pre-requisite for this to be feasible is the correctness of the R-wave markers on the ECG trace. As shown in the Results section this assumption did not always prove to be correct and, in order to compensate the effect of this phenomenon, the images acquired in those cycles in which a certain reference to determine the phase error was missing, were excluded from the analysis. Despite this correction, in 4 patients for the apical view and in 3 for the parasternal view the mean phase error exceeded the threshold of 0.1. As evidenced by our analysis, one possible explanation for this difficulty may lie in cardiac rhythm instability. In these conditions, the constant variability of the RR interval deprives the algorithm of a unique reference for its functioning in this task. The same difficulty would also appear at treatment planning when a cardiac 4D-CT has to be acquired. If the heart rhythm is not stabilized, it will not be possible to obtain adequate CT images for treatment planning. In order to overcome this limitation, one could, in particular in patients with a device, enhance the negative dromotropic therapy and/or increase the pacing rate to try to regularize the frequency. The *rate smoothing* algorithms could also be useful for this purpose (19). Moreover, once the image acquisition and the algorithm operation are optimized, it will not be strictly necessary to have a high-quality ECG trace with correct R-wave markers, because the algorithm operation is independent from the ECG trace, which is used for the purposes of the study to have a reference in the calculation of the phase error, and not for the intrinsic operation of the system.

For the evaluation of score B (magnitude of maximum displacement and error on the calculation of displacement) the quality of the acquired images allowed a positive scoring in most of the patients. In contrast to what was reported for the evaluation of score A, there was no statistically significant influence on the performance of the algorithm by R-R cycle instability. This being said, due to the small number of patients enrolled in this feasibility study, strong conclusions cannot be drawn. In contrast to the evaluation of score A, it should be mentioned that there was no reference (ground-truth) of the true cardiac displacement in the study that would allow to evaluate the error in the actual performance of the algorithm. The quality of the acquired images was adequate for most of the patients, as demonstrated by the fact that the magnitude of the calculated maximum displacements was plausible and the calculated error on the displacement was in line with what observed on the validation set. In order to plan a future clinical application of the system studied, it will be necessary to evaluate the accuracy of the amount of displacement calculated by the algorithm with respect to the actual cardiac displacement monitored using a reference method. Further studies are planned to answer this question.

A further consideration to be made concerns the visual assessment of the quality of the images (score C). Compared to the images normally used for clinical purposes, the quality of the images automatically acquired by the system in our study is on average significantly lower. This is because the data displayed in the prototype user interface are the raw images and none of the usual visualization post-processing techniques (e.g., frame averaging or speckle reduction) have been implemented. Future versions of the prototype will instead include these visualization tools. This being said, the purpose of the acquisition is not to obtain images of diagnostic quality, but adequate to be interpreted automatically by the algorithm and to ensure that the operator can check that the heart is visible in the picture. This is the reason why in the evaluation of score C, a less restrictive criterion was used, accepting as sufficient even the visualization of a single cardiac structure. Since the visualization of a specific cardiac structure is not necessary for the functioning of the algorithm, thanks to the machine-learning approach, even images which are not perfectly interpretable by the human eye are acceptable, extending the audience of patients in which this method can be applied also to patients with a non-optimal acoustic window if evaluated with standard criteria. Also, in view of this, it is not surprising that the ablative target itself does not necessarily need to be identified by ultrasound. Being a cardiac target, monitoring of the organ itself should theoretically ensure sufficient accuracy.

Assuming that the evaluation of the displacement is accurate, for the system to be able to guide a radiotherapy treatment it will have to be able to recognize precisely the phase of the cardiac cycle in which each image is acquired, calculate accurately the displacement comparing images acquired in similar phases of different cardiac cycles and, to do this, at least one cardiac structure will have to be always visible and the heart should not disappear with respiration. Based on this consideration, we have defined as maximal that image quality that satisfies all 4 points of the multi-parametric score. No significant differences were found in the clinical and demographic characteristics of patients with maximal image quality compared to those with lower quality, except for those factors that limit the regularity of the cardiac cycle, as previously discussed. None of the patient's physical characteristics were found to be significantly associated with lower image quality, although the limited sample size does not allow any definite conclusion in this matter. Based on our results it is likely that an echocardiographic system could be of clinical utility to guide radiotherapy treatment in most patients (i.e., about 80%).

A possible limitation of the use of this system during the delivery of therapy could be represented by the interaction between the probe positioned on the chest and the radiant beam. Future studies have already been planned to verify this risk.

If further studies will confirm the functioning of this system, it can be hypothesized that its clinical application could lead to significant advantages for STAR. With a real-time system for heart monitoring available, the need to increase the target volume to compensate for cardio-respiratory movements could be limited. In addition, a precise gating or tracking could be done without the need and limitations of a fiducial marker. This could further reduce the safety margins to be applied and perhaps reduce treatment times, particularly in case of respiratory gated delivery for radiotherapy with heavy particles such as protons and carbon ions (9, 10, 12, 20). A further usefulness of this system could consist in increasing the safety in the treatment phase, by controlling in real time the cardiac cycle and heart movements and allowing to interrupt the delivery of the radiant beam in case of anomalies on each of these two factors with extremely short reaction times. For the purposes of clinical applicability, it will also be important to develop and optimize the communication and integration between the ultrasound system and the systems commonly used for treatment planning and radiation beam delivery. Finally, further studies on a larger population will be needed to confirm the feasibility of this system and to optimize its operation on the widest possible spectrum of patients with different physical and clinical characteristics.

## Conclusions

The results of the present study provide the proof-of-concept for the feasibility of an automatic ultrasonographic image acquisition system associated with an AI algorithm for real-time monitoring of cardiac motion in patients with a history of VAs. Although further studies are needed before this system can be applied to clinical practice, the possibility of real-time, non-invasive monitoring of cardiac position would lead to a significant improvement in the quality and safety of stereotactic radiotherapy treatment for patients with VAs.

## Data Availability Statement

The raw data supporting the conclusions of this article will be made available by the authors, without undue reservation.

## Ethics Statement

The studies involving human participants were reviewed and approved by Comitato Etico Pavia, Fondazione IRCCS Policlinico San Matteo, Pavia, Italy. The patients/participants provided their written informed consent to participate in this study.

## Author Contributions

AG, SC, JG, and TB designed and developed the prototype used for the acquisitions and the AI algorithm. MC, VD, AG, SC, and JG carried out the acquisitions. MC, AG, and JG performed the analyses on the acquired data. MC wrote the draft of this manuscript. All authors discussed the results and approved the final version of the manuscript.

## Funding

This publication is part of a project that has received funding from the European Union's Horizon 2020 research and innovation program under grant agreement No 954783.

## Conflict of Interest

The Fondazione IRCCS Policlinico San Matteo, Pavia, Italy, stipulated an agreement with EBAMed SA, Geneva, Switzerland to conduct the study and exchange data. SC, JG, TB, and AG are employed by EBAMed SA. MC has a consultancy agreement with EBAMed which began after the conduction of this study. The remaining authors declare that the research was conducted in the absence of any commercial or financial relationships that could be construed as a potential conflict of interest.

## Publisher's Note

All claims expressed in this article are solely those of the authors and do not necessarily represent those of their affiliated organizations, or those of the publisher, the editors and the reviewers. Any product that may be evaluated in this article, or claim that may be made by its manufacturer, is not guaranteed or endorsed by the publisher.
